# Small *de novo* CNVs as biomarkers of parental exposure to low doses of ionizing radiation of caesium-137

**DOI:** 10.1038/s41598-018-23813-5

**Published:** 2018-04-12

**Authors:** Emília Oliveira Alves Costa, Irene Plaza Pinto, Macks Wendhell Gonçalves, Juliana Ferreira da Silva, Lorraynne Guimarães Oliveira, Alex Silva da Cruz, Daniela de Melo e Silva, Cláudio Carlos da Silva, Rinaldo Wellerson Pereira, Aparecido Divino da Cruz

**Affiliations:** 1 Pontifical Catholic University of Goiás, Department of Agricultural and Biological Sciences, Genetics Master’s Program, Replicon Research Group, Rua 235, n. 40, Bloco L, Área IV – S. Universitário, CEP 74605-050 Goiânia-GO, Brazil; 2 Human Cytogenetics and Molecular Genetics Laboratory, Health Secretary of Goiás State, Goiânia-GO, Brazil; 3 Catholic University of Brasilia, Genomic Sciences and Biotechnology Graduate Program, Brasilia-DF, Brazil; 4 University of Brasília, Biotechnology and Biodiversity PhD Program, Brasilia-DF, Brazil; 5 State University of Goiás, UnU Goiania, Goiânia-GO, Brazil; 6 Federal University of Goiás, Graduate Program in Genetics and Molecular Biology, Institute of Biological Sciences, Goiânia-GO, Brazil

## Abstract

The radiological accident in Goiania in 1987 caused a trail of human contamination, animal, plant and environmental by a radionuclide. Exposure to ionizing radiation results in different types of DNA lesions. The mutagenic effects of ionizing radiation on the germline are special concern because they can endures for several generations, leading to an increase in the rate of mutations in children of irradiated parents. Thus, to evaluate the biological mechanisms of ionizing radiation in somatic and germline cells, with consequent determination of the rate mutations, is extremely important for the estimation of genetic risks. Recently it was established that Chromosomal Microarray Analysis is an important tool for detecting wide spectra of gains or losses in the human genome. Here we present the results of the effect of accidental exposure to low doses of ionizing radiation on the formation of CNVs in the progeny of a human population accidentally exposed to Caesium-137 during the radiological accident in Goiânia, Brazil.

## Introduction

Unexpected events led to a serious radiological accident involving Caesium-137 in September of 1987 in Goiânia, Brazil. Consequently, people and the environment were contaminated with radionuclide and exposed to ionizing radiation (IR) during a two-week period in which the accident had not been discovered yet. A total of 249 people was exposed to IR with absorbed doses ranging from near zero up to 7 Gy. For an extensive review of the accident, the readers are referred to da Cruz *et al*. & IAEA^1,2^. Despite being disastrous, the accident provided a unique opportunity to study the biological effects of ionizing radiation-induced mutations in humans.

The genetic health of people accidentally exposed to Caesium-137 has been continuously biomonitored in Goiânia since the accident. To date, most of the genetic biomarkers of exposure available were examined for the cohort of families accidently exposed to IR. Currently, reports are accessible for chromosomal aberrations, glycophorin A mutations micronucleus assay, *HPRT* test, somatic chromosomal translocation using FISH germline mutation rate in STRs, assessment of BCL2/J(H) translocations, and date for serological autoimmune markers^2–9^.

At a cellular level, exposure of biomolecules to IR results in molecular lesions. When human DNA is exposed to IR, immediate damage is mostly of a result of either double strand breaks (DSBs) or single strand breaks (SSBs). The damage is enzymatically repaired, resulting in either the removal of damaged bases or the re-joining of strand breaks. However, incorrect repair of the DNA damage leads to mutations that contribute to adverse stochastic effects. The nature of cells and tissues defines their radiosensitivity, which is the highest in highly mitotic or undifferentiated cells. Thus, germline cells are highly sensitive to IR exposure, and mutations in germ cells could increase the mutation rate in the offspring of irradiated parents^10,11^.

Lately, chromosomal microarray analysis (CMA) has been recognized as a powerful tool to detect gains and losses of the human genome and has been recommended as the new standard test to accurately detect chromosomal variations of different types, sizes, and genomic locations at higher resolution than karyotyping and conventional fluorescent *in situ* hybridization (FISH)^12,13^. CMA is able to detect both losses and gains of copy number variants (CNV) based on the distribution of millions of SNP markers across the whole genome^14^. A CNV is defined as gain or loss of a DNA segment ≥50 pb broadly scattered through a given genome^15–17^. On the other hand, some authors only apply the definition of CNV to DNA segments ≥1 kb^18^.

The identification of CNVs was facilitated by the advent of new technologies that allowed high-resolution genomic analysis by oligonucleotide microarrays (14). Investigating CNVs revealed their crucial role in population diversity, both as the reason for genetic variability and the etiological causes of human diseases. The frequency with which CNVs are formed suggests a high rate of *de novo* mutations that can be transmitted by Mendelian over generations^19–21^.

The mechanisms subjacent to the formation of CNVs have gained much attention from researchers. It has been postulated that both DNA replication and homologous recombination are important events in the origin of CNVs^15,21,22^. Due to their nature, CNVs could be useful biomarkers of exposure to assess potential health risks related to environmental mutagens. The frequency of *de novo* CNVs increased in cells exposed *in vitro* to ionizing radiation^23^. Thus, the rate of CNV formation could be useful to discriminate both somatic as well as germline mutation in humans. Moreover, the identification of inherent biomarkers of radiation exposure is a priority in radiation research in the genome era^24^. The usefulness of the rate of *de novo* CNV formation as a biological marker for human exposure to IR remains mostly unknown and must be investigated^25^.

In general, studies of CNV have reported findings ≥500 kb. Some population studies have been generated to evaluate the role of larger CNVs. Itsara *et al*.^26^, estimated the frequency of large CNVs in the general population using the data of about 2,500 people, focusing on hotspots prone to recurrent mutations. The authors found CNVs ≥500 kb and ≥1 Mb in up to 1–2% of individuals. To date, studies describing the germline mutation rate and paternal age at conception for small CNVs have not been reported. The current study reports the results of germline mutation rates using CNVs from the progeny born to parents accidentally exposed to very low doses of IR during the radiological accident with Caesium-137 in Goiania (Brazil) in 1987. In this context, the authors aimed to demonstrate the potential of CNVs germline mutations to be retrospectively used as biomarkers of exposure to IR in human populations.

## Results

The mean parental ages at conception were 31.4 and 32.3 for fathers, and 26.4 and 29.6 for mothers, in cases and controls, respectively. The exposed population incurred individual doses of ≤0.2 Gy. The average ages of the F1 generations were 14.5 and 12.3 for cases and controls, respectively. Tables 1 and 2 summarize some of the data in the current study.

**Table 1:** Summary of data from case and control groups for both parental and F1 generation included in the study of induced germline mutation in the offspring of people accidently exposed to low doses of ionizing radiation from Caesium-137 in Goiânia (Brazil).

Groups	Family	Number of CNVloss	Number of CNVgain	Paternal Age	Maternal Age	F1’s Age	F1’s Sex
Control	CMA001-1F	39	1	40	36	9	Female
	CMA002-1F	34	1	22	21	9	Male
	CMA003-1F	20	1	26	26	25	Female
	CMA004-1F	32	1	30	31	10	Female
	CMA005-1F	29	2	42	31	5	Female
	CMA006-1F	24	3	37	35	6	Male
	CMA007-1F	27	3	47	36	9	Male
	F09-3F	19	2	19	21	25	Male
Total	—	224	14	—	—	—	—
Case	F04-2F	18	3	27^a^	27	20	Male
	F06-2F	26	3	35^a^	26	9	Male
	F07-1F	26	5	54	24^b^	19	Male
	F07-4F	30	2	56	26^b^	17	Female
	F08-2F	13	5	18	20^b^	8	Male
	F09-2F	9	1	24^c^	26^c^	20	Female
	F10-2F	16	1	21	24^b^	2	Female
	F12-2F	43	3	31	30^b^	3	Male
	F15-2F	26	0	18^a^	27	16	Male
	F18-2F	36	11	47^a^	30	18	Male
	F21-2F	18	2	38	27^b^	20	Female
	F22-2F	42	2	29	31^b^	20	Female
	F22-3F	45	2	32	34^b^	17	Female
	F22-4F	25	3	33	35^b^	16	Male
	FAD-24F	29	4	21^a^	19	12	Female
	FAD-25F	20	4	18^a^	16	15	Female
Total	—	422	51	—	—	—	—

CNVloss: genomic losses; CNVgain: genomic gains. ^a^Exposed father; ^b^exposed mother; and ^c^both parents exposed.

**Table 2:** Descriptive data from case and control groups for both parental and F1 generations included in the study of germline mutation induction detected in the progeny of people accidentally exposed to ionizing radiation from Caesium-137.

Generation	Variables		Controls	Cases
Parental	^n^		16	24
	Age Range		16 to 56	19 to 47
	Average Age at Conception (±SD)	Fathers	32.3 (10.1)	31.4 (12.3)
		Mothers	29.6 (6.3)	26.4 (5.1)
	Dose (Gy)		0	0.2
F1	n		8	16
	Age Range		5 to 25	2 to 20
	Average Age (±DP)		12.3 (8.0)	14.5 (6.0)
	Sex Ratio (M/F)		4/4	9/7
	Mean ${{\rm{MR}}}_{{{\rm{CNV}}}_{\text{gain}/\text{ger}}}$ (SD)		1.5 × 10^−5^ (±9.7 × 10^−6^)	2.2 × 10^−5^ (±1.23 × 10^−5^)
	Mean ${{\rm{MR}}}_{{{\rm{CNV}}}_{\text{gain}/\text{ger}}}$ (SD)		6.2 × 10^−6^ (±6.4 × 10^−6^)	1.0 × 10^−5^ (±1.3 × 10^−5^)
	Mean ${{\rm{MR}}}_{{{\rm{CNV}}}_{{\rm{\beta }}}}$ (SD)		2.2 × 10^−5^ (±1.1 × 10^−5^)	3.2 × 10^−5^ (±1.9 × 10^−5^)

SD: Standard Deviation; M: Males; F: Females; ger: Generation; β: Burden.

No significant differences between cases and controls in the number of mutational events, either gains or losses, were observed. The differences arose when the data were transformed to the proportion of the gained or lost genome as a function of the total size of CNVs (Equation 1), where genomic losses and gains were higher in the offspring of exposed parents, corresponding to an increase of 1.5x for losses and 1.6x for gains when compared to the control group. In this context, the burden of the germline mutation rate in CNVs (MR_CNVβ_) increased about 50% in the genome of children whose parents were exposed to low doses of IR when compared to the offspring born to parents with no history of IR exposure from the same population (Goiânia).

With respect to the total number of CNVs, we identified 473 and 238 CNVs for case and control groups, respectively. Distribution and characterization of CNVs between the groups can be found in Table 3. In the current study, the largest CNV size was 393 kb. All observed CNVs in the current study were included in the statistical analysis. Genomic losses ranged from 1 kb to 147 kb, while gains ranged from 1 kb to 393 kb. The total size of the gains and the losses in the genomes of the exposed group were 1,892 kb and 4,103 kb. However, in the control group, gains added up to 579 kb and losses up to 1,428 kb (Table 3). Using the tools intrinsic to ChAS® (Affymetrix, USA), it was possible to assign the parent of origin (POR) for 60% (428/711) of all called CNVs, distributed as follow: 133 and 285 CNVs for control and cases, respectively. With respect to POR, 71 CNVs we transmitted by fathers as well as 81 transmitted by mothers to their offspring, considering the transmission by the exposed parents in the group of cases cohort.

**Table 3:** Distribution of CNVs between cases and controls observed in the study of the impact of accidental exposure to low doses of ionizing radiation on the frequency of germline mutations in the progeny of people exposed during the Goiânia radiological accident with Caesium-137. ΣT_CNV_: Total size of CNVs (kb); na: not applicable.

Group	CNV	Gain	Loss	Total
Case	Interval (kb)	1 to 393	1 to147	1 to 393
	n	51	422	473
	Average of CNV Size (kb)	37.1 (±58.8)	9.7 (±13.8)	na
	ΣT_CNV_ (kb)	1.892	4.103	5.995
Control	Interval (kb)	1 a 140	1 a 85	1 a 140
	n	14	224	238
	Average of CNV Size (kb)	41.4 (±48.2)	6.4 (±8.5)	na
	ΣT_CNV_ (kb)	579	1.428	2.007

**Figure 1:**
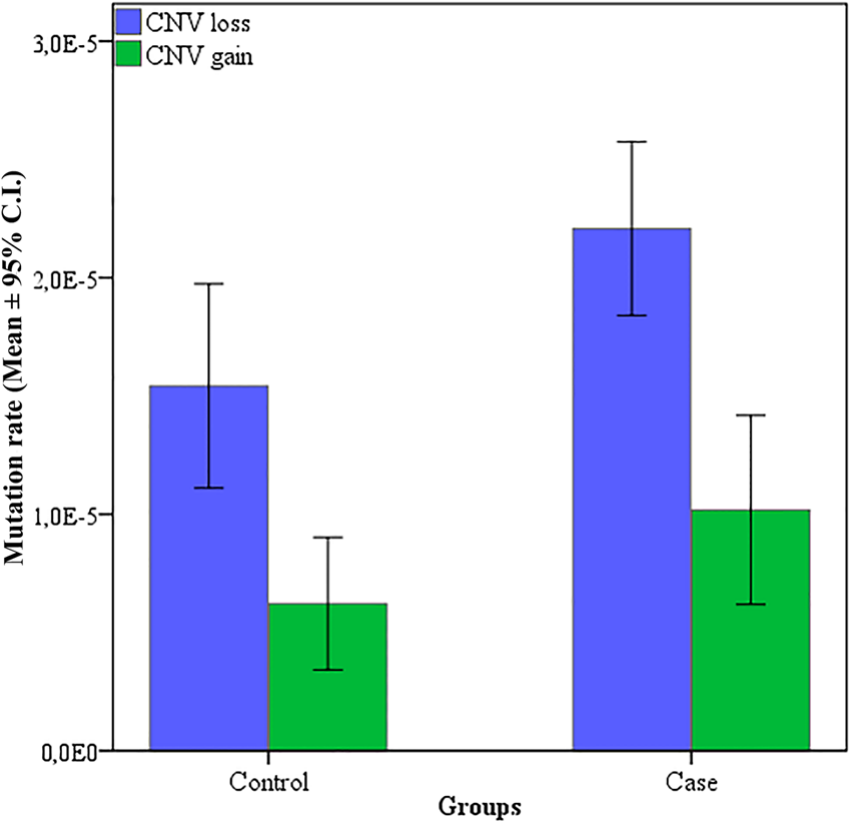
Average of germline mutation rates in CNVs from the progeny of controls and cases who were accidentally exposed to low doses of ionizing radiation from Caesium-137 in Goiânia (Brazil). CNVloss: genomic loss; CNVgain: genomic gain.

**Figure 2:**
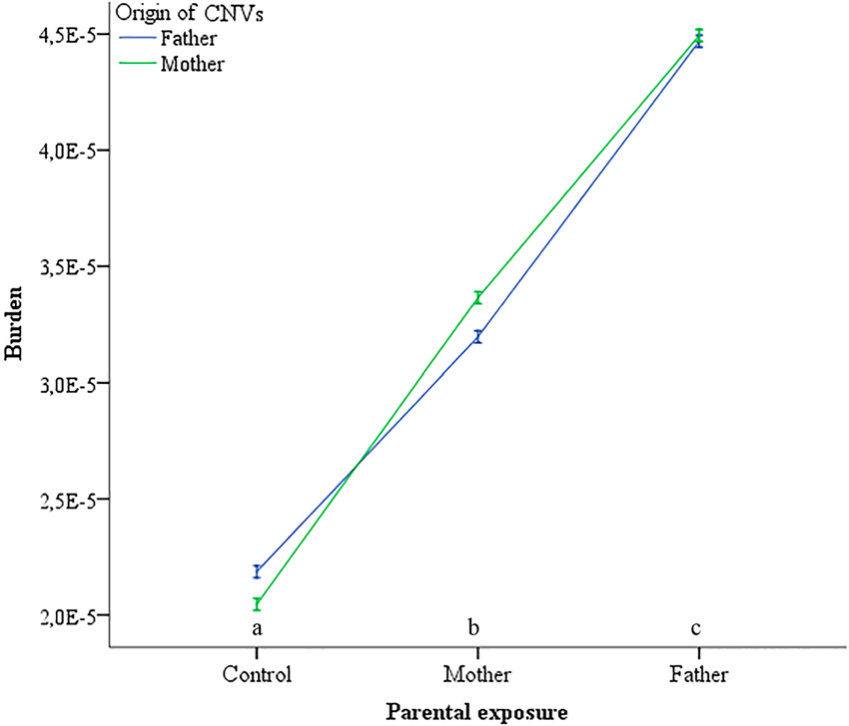
Burden of CNVs as a function of parental exposure and origin of CNVs for cases and control groups in the study of the effect of low absorbed doses of IR on the rate of germline mutations. Letters indicate significant differences among parental exposure at a significance level of 5%.

Figure 1 shows the differences in the distribution of MR_CNV_ between case and control groups. The Mann-Whitney U test indicated the difference for genomic losses was statistically significant (p < 10^−3^). However, no statistical significance for genomic gains was observed between the studied groups (p = 0.76). The results of Two-way ANOVA followed by the Tukey’s Test *a posteriori* were performed to compare the contribution of parental exposure and the parent of origin (POR) to the burden of the *de novo* CNV in their progenies showed significant differences among parental exposure (F = 61,03; p < 0,0001). On the other hand, there was no significant difference (F = 0,00; p = 1,00) between the origin of the CNVS and POR (Fig. 2).

The binomial test showed the genome was affected differently between cases and controls, with respect to genomic losses (χ^2^ = 39.06; p < 0.00001) and gains (χ^2^ = 284.8; p < 0.00001). A similar statistical significance was observed even if the unequal cohort size was taken into account for genomic losses (χ^2^ = 378.99; p < 0.00001) and gains (χ^2^ = 5.7; p = 0.02).

**Figure 3:**
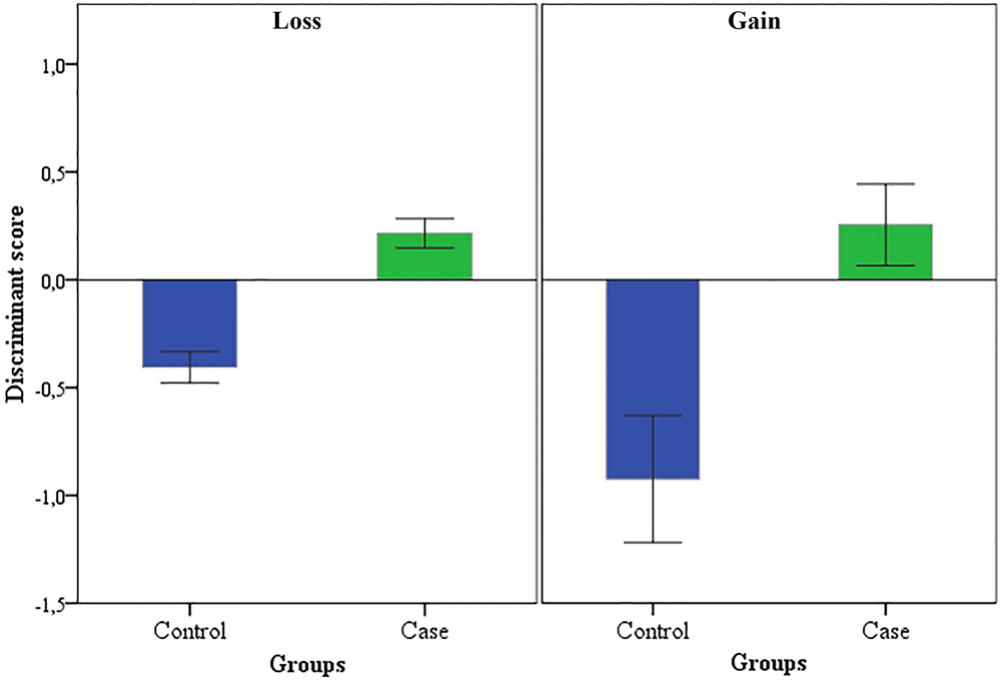
Discriminant function analysis of predictors: number of paternal meiosis, mean parental age at conception, and number of marker in a CNV on the germline mutation rate of genomic losses and gains for cases and controls observed in the progeny of a population exposed to low doses of ionizing radiation and a control population from Goiânia (Brazil).

Discriminant function analysis was used to determine which variables discriminated between exposed and control groups. In the current study, the best three predictors to discriminate the groups were number of paternal meiosis, mean parental age at conception, and number of markers in a CNV. The differences were statistically significant for both genomic gains (p = 0.02; r^2^ = 0.17) and losses ((p < 0.001; r^2^ = 0.08). The scores of linear discrimination are depicted in Fig. 3.

**Figure 4:**
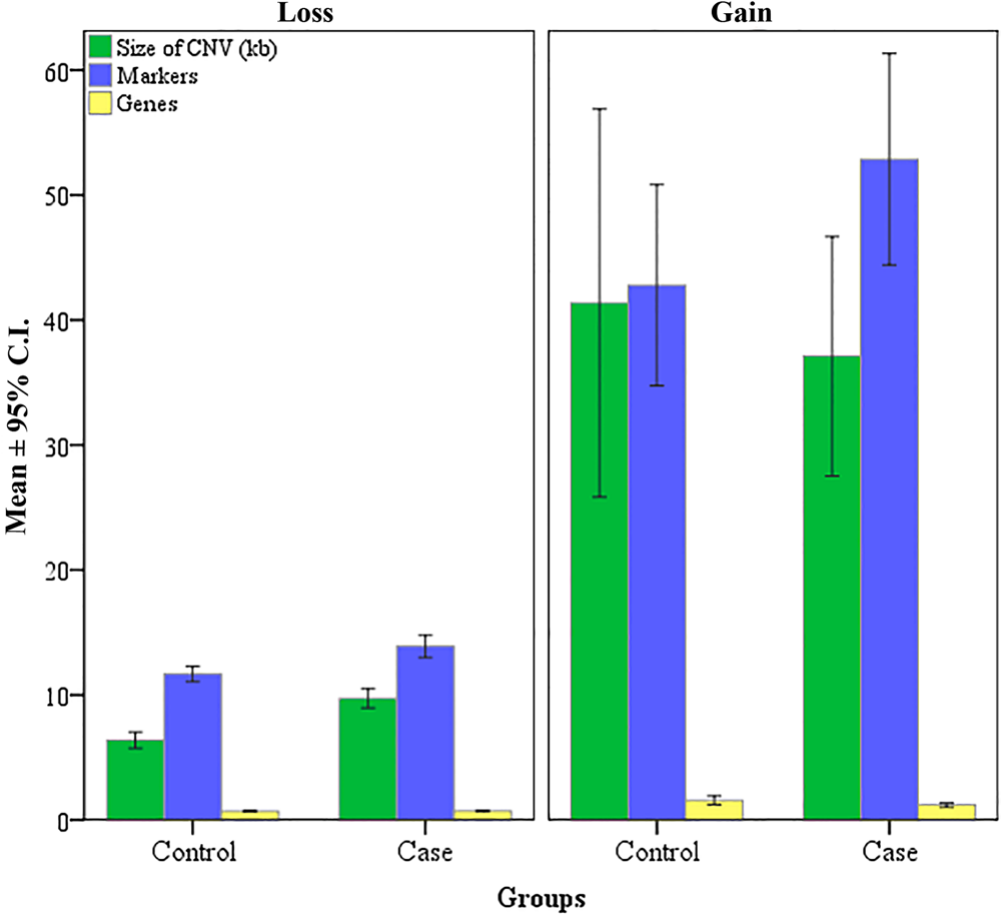
Distribution of CNV size, number of markers and number of genes in the CNVs for both control and exposed groups to low doses of ionizing radiation of Caesium-137.

The Mann-Whitney U was used to test the differences in the distribution of CNV size, number of markers and number of genes within the CNVs (Fig. 4). There were no statistical differences for the tested variables between the studied groups, in the current study.

**Figure 5:**
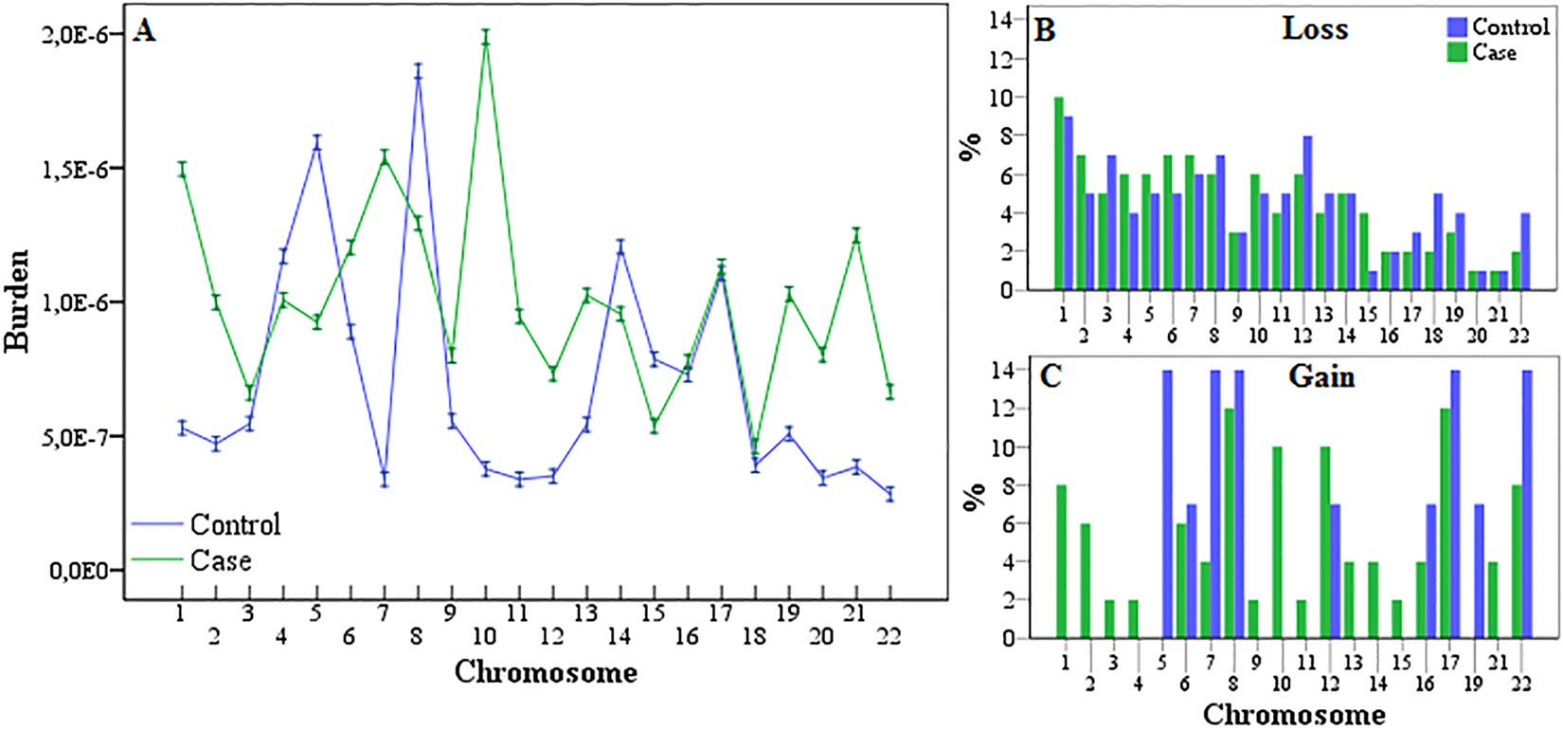
Results of the Mann- Whitney U test of the *burden* of CNVs (**A**) and the total of CNVs per chromosome for genomic losses (**B**) and genomic gains (**C**) between case and control groups in the study of the induction of germline mutation due to parental exposure to ionizing radiation of Caesium-137.

In order to compare the burden of *de novo* CNVs (Fig. 5A) and the proportion of chromosomal genome affected by the CNVs (Fig. 5B,C) per chromosome for both losses and gains, we applied the Mann-Whitney U test. Statistically significant differences between case and control groups were observed for chromosomes 1, 2, 7, 10, 11, 12, 19, and 20 (Table 4).

**Table 4:** Rate of germline mutation per chromosome in the offspring of parents exposed to ionizing radiation of Caesium-137 and the control group.

Chromosome	Control		Case		P value
	Average	SD	Average	SD	
1	5.3E-07	8.3E-07	1.5E-06	2.5E-06	0.04
2	4.7E-07	4.8E-07	1.0E-06	1.1E-06	0.04
3	5.5E-07	5.3E-07	6.6E-07	7.3E-07	0.51
4	1.2E-06	1.3E-06	1.0E-06	9.7E-07	0.98
5	1.6E-06	1.5E-06	9.2E-07	8.7E-07	0.11
6	8.9E-07	2.0E-06	1.2E-06	1.5E-06	0.07
7	3.4E-07	3.2E-07	1.5E-06	2.6E-06	0.003
8	1.9E-06	3.7E-06	1.3E-06	2.4E-06	0.81
9	5.6E-07	4.9E-07	8.0E-07	9.0E-07	0.63
10	3.8E-07	3.3E-07	2.0E-06	6.2E-06	0.007
11	3.4E-07	4.9E-07	9.5E-07	2.0E-06	0.02
12	3.5E-07	3.5E-07	7.3E-07	7.7E-07	0.03
13	5.4E-07	5.7E-07	1.0E-06	1.2E-06	0.06
14	1.2E-06	2.2E-06	9.6E-07	8.4E-07	0.27
15	7.9E-07	6.2E-07	5.4E-07	4.7E-07	0.35
16	7.3E-07	9.3E-07	7.8E-07	8.5E-07	0.44
17	1.1E-06	9.4E-07	1.1E-06	1.4E-06	0.47
18	3.9E-07	4.1E-07	4.6E-07	2.7E-07	0.23
19	5.1E-07	5.4E-07	1.0E-06	6.3E-07	0.04
20	3.4E-07	8.5E-08	8.0E-07	4.8E-07	0.04
21	3.9E-07	6.4E-08	1.2E-06	1.4E-06	0.99
22	2.8E-07	2.3E-07	6.6E-07	1.1E-06	0.39

**Figure 6:**
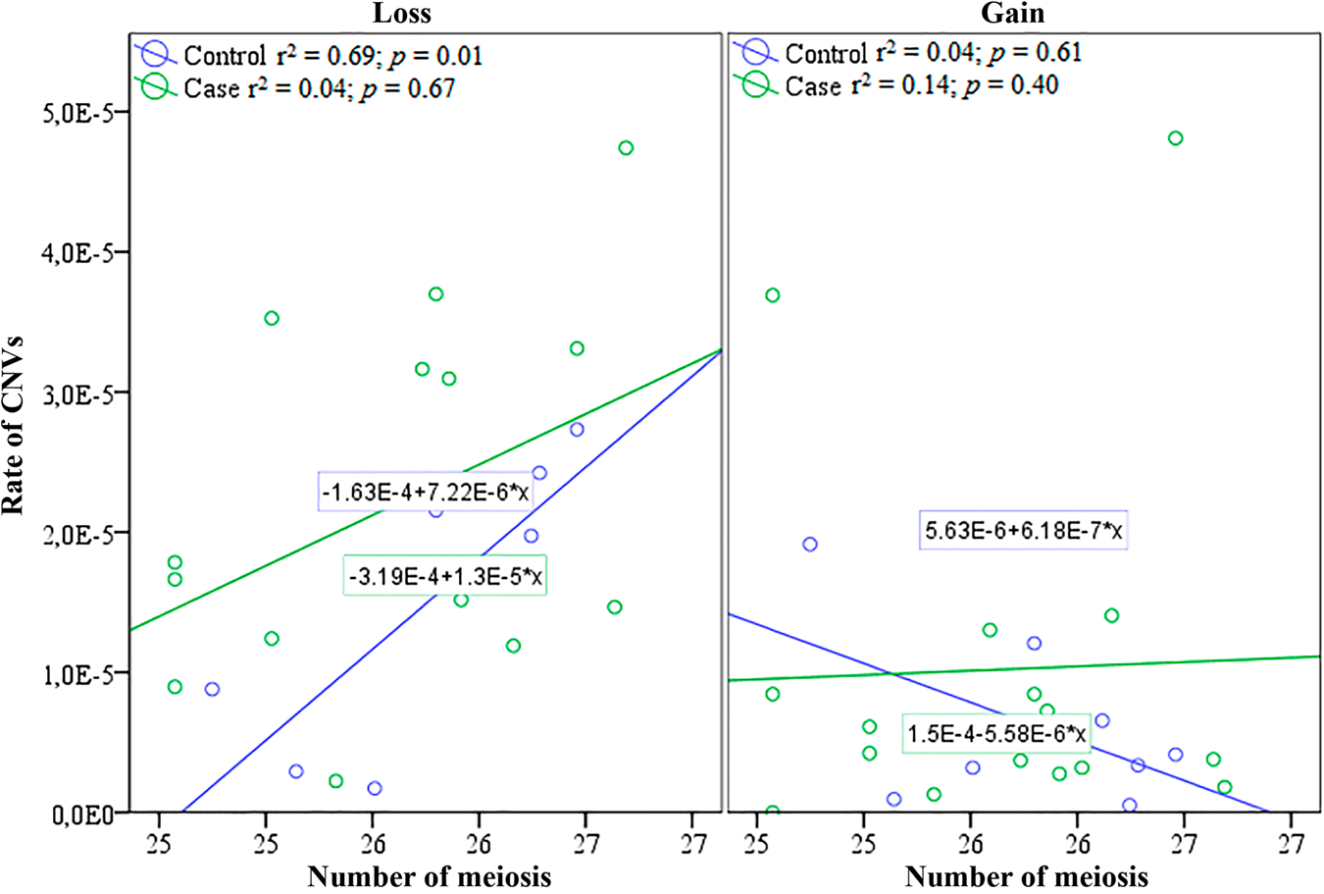
Spearman**’**s correlation test applied to the number of paternal meiosis at the time of conception and the burden of CNV germline mutation in F1 of controls and cases, corresponding to children born to fathers accidentally exposed to low doses of ionizing radiation of Caesium-137 in Goiânia (Brazil).

Spearman’s correlation test was applied to the number of paternal meiosis at the time of conception and the burden of CNV germline mutations in the progenies of case control population (Fig. 6). For the exposed pupation, the test was performed using only the fathers exposed to IR from 137Cs. Positive and statistically significant association was observed for genomic losses in the control group. However due to the paternal exposure, the significance was lost for genomic gains in the exposed population. Microduplications were not statistically significant for neither case nor control populations.

**Figure 7:**
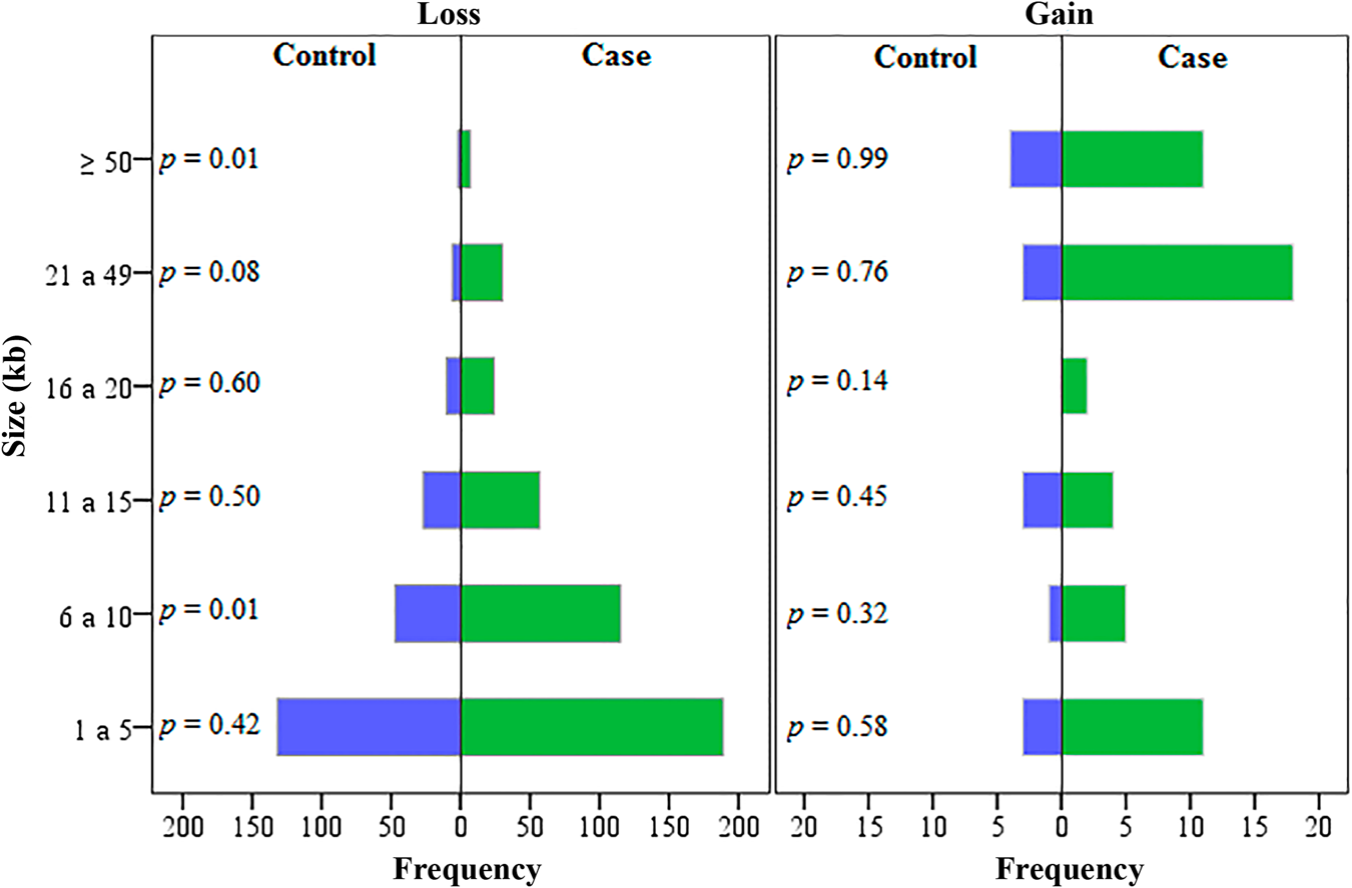
Frequency distribution of different classes of CNV sizes (kb) between controls and exposed groups for both genomic losses and gains, in the study of the effect of ionizing radiation in the induction of germline mutations CNV.

**Figure 8:**
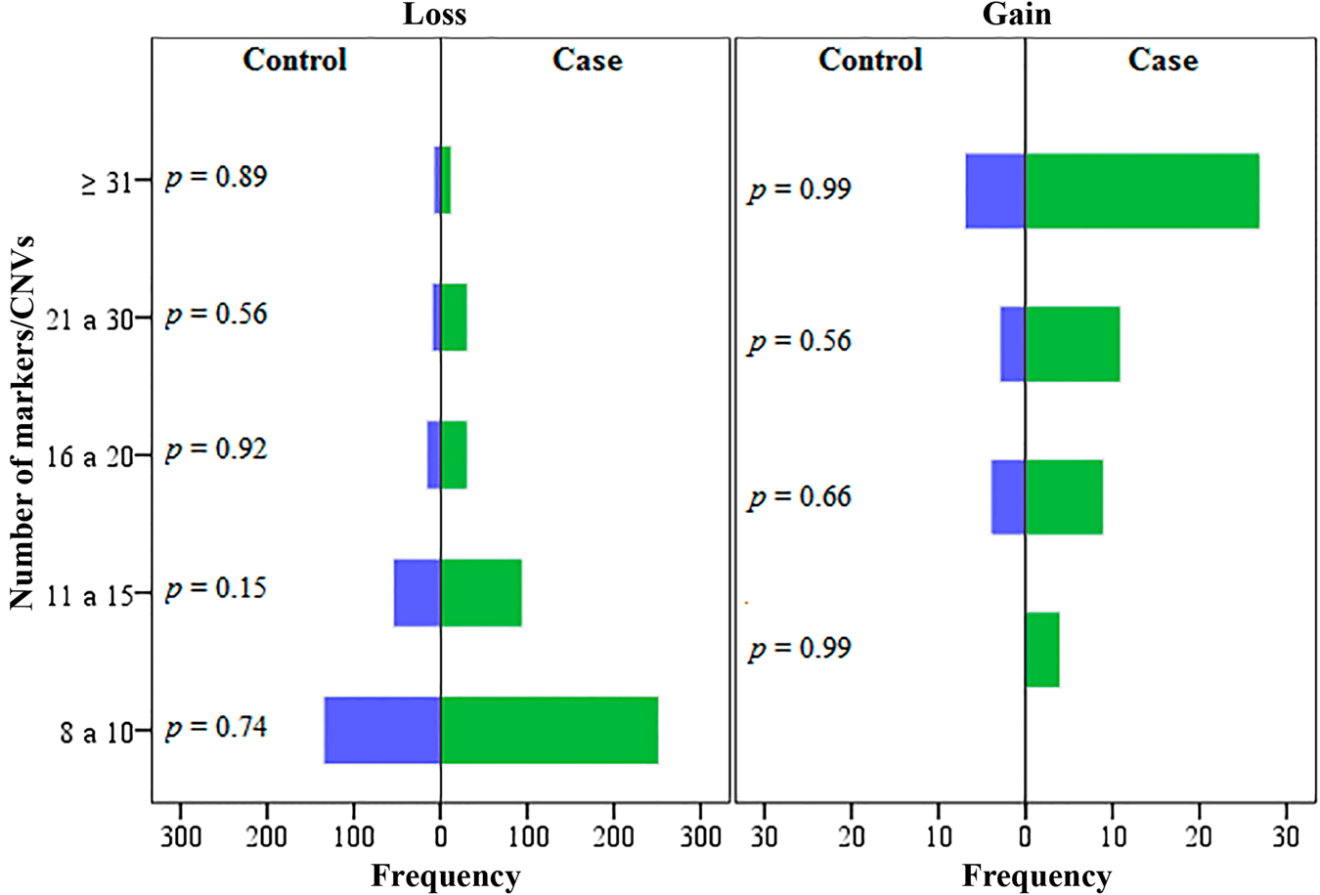
The χ^2^ post-hoc test showed no statistical differences in the frequency distribution of the classes of CNV according to the number of markers between cases and controls in the study of the effect of ionizing radiation in the induction of germline mutations CNV.

In order to test for the differences of the frequency of CNV size (kb) and the number of markers/CNV for both genomic losses and gains, the χ^2^ post-hoc test was used. CNV sizes for genomic losses ranging from 6 to 10 kb and ≥50 kb from cases were statistically different from the control group. On the other hand, no differences were observed for the genomic gains with respect to their size (Fig. 7). Similarly, there were no statically significant differences for the number of markers/CNV between cases and controls (Fig. 8).

## Discussion

The vast majority of studies about CNVs in humans are focused on DNA segments ≥500 kb^27,28^. To the best of our knowledge, the current study is the first to report the use of genomic gains and losses of small CNVs (≤500 kb) to estimate *de novo* human germline mutation rates in the offspring of a parental population accidentally exposed to ionizing radiation of caesium-137. In the current study, the number of mutational events weren’t statistically different between cases and controls. However, when, the data were transformed to the proportion of genomic gain or loss for an individual genome as a function of the total size of CNVs, the difference emerged. Thus, given the potential deleterious effect of acute whole-body external exposure to IR on the germ cell lines, the genome of children born to parents exposed to low doses rates of ^137^Cs gamma radiation exhibited about 3x more of global genomic loss and gain when compare to the offspring born from the control population (Table 3).

There is a considerable worldwide effort to estimate the mutation rates of CNVs in humans. Initial studies estimated CNV mutation rate at 2.5 × 10^−6^ per locus per generation^29,30^. Whole genome analysis for 400 parent-child trios detecting CNVs ≥100 kb reported a *de novo* mutation rate of 1.2 × 10^−2^ variants per generation^27^. A higher mutation rate of 3.6 × 10^−2^ CNVs per generation was observed in individuals with intellectual disabilities^31^. In the current study, CNV mutation rates for both gains and losses were defined as burden. The burden of CNV mutations in the germ cells was 2.2 × 10^−5^ and 3.2 × 10^−5^ for control and exposed populations, respectively, thus, increasing 1.5x the mutation burden in the offspring born to parents exposed to ionizing radiation when compared to unexposed controls. To date, there are no reports on the rate of CNV germline mutations due to population exposure to IR in humans. Nevertheless, our findings are in agreement with the CNV mutation rate reported by Campbell & Eichler^30^ in their extensive review of the properties and rates of germline mutations in humans. For advanced understanding on the genome-wide effects of ionizing radiation on mutation induction in the mammalian germline the authors suggest the work of Adewoye *et al*.^11^. With respect to the transmission of *de novo* CNVs based on parental exposure, we report the highest rate was observed for exposed fathers and mothers when compared to the controls. Moreover, the paternal contribution was higher than the maternal contribution to the burden of CNV in their offspring within the group of exposed parents.

It has been well established that mutation rates differ within in between families^32,33^. Reports on comparing *de novo* mutations in sibs of multisibling families in which one sib was conceived prior to a parent being exposed and the other after has ben helpful to understand how the environment interacts with the inherited genome. For both human and animal models exposed to radiation, most studies reported the post exposure offspring had higher significantly germline mutation rates when compared with pre-exposure offspring^34^. On the other hand, epidemiologic studies have failed to demonstrate a significantly increased risk of genetic defects or congenital malformations in children born to parents treated with radiotherapy^35^. Curiously, in the current study, for one family (F09) we had siblings conceived before (F09-3F) and after (F09-2F) both parents were accidentally exposed to IR. Interestingly, the child born after the parental exposure showed fewer CNV loses and gains (Table 1) despite being born 5 years after her brother. Moreover, this child has the smallest mutation rate in the entire cohort. Although, nothing could be concluded based on a single observation, our finding warrants the need for further evaluation of the potential effect of genome and environmental interactions. Thus, the discussion about the impact of human exposure to low doses of IR and its consequence on the germline mutation rate in the offspring conceived after parental exposure is still far from over.

The variables namely number of paternal meiosis, mean parental age and number of markers distributed in the CNVs, were the strongest predictors to contribute to the burden of CVN mutations in the offspring and allowed the discrimination between exposed and unexposed groups. Regarding the recurrence of CNVs, no hotspots in the chromosomes were identified, confirming that induced CVNs fit the random-effect model of radiation exposure. This observation was expected due to the random deposition of radiation energy on biological systems. Here, we report that the mutation rate of genomic losses was greater than gains and the effect of paternal age was better discriminated when analysing the frequency of losses. This observation is supported by previous reports about the increase of mutations frequencies and rates in relation to the advancing age of fathers^36^.

The common knowledge that most radiation-induced mutations are deletions is nothing new. Genetic analysis studies of specific loci reported that radiation exposure of mouse germ cells induces deletions of different sizes, more frequently observed when animals have incurred high absorbed doses of radiation^37^. However, a central question remains about the induction of small deletions following human germ cells exposure to low absorbed doses of ionizing ration^38–41^. Despite the vast amount of information accumulated and the technological advances in the field, the knowledge about the mechanism of DNA repair associated with DSBs and SSBs subjacent to the induction of large and small deletions by ionizing radiation in human models have been consolidated^37,41–45^.

NAHR and MMBIR are considered as responsible for generating deletions of varying sizes, which is consistent with independent data on genomic disorders associated with recurrent deletions of different lengths (mostly by NAHR) and other disorders associated with non-recurrent and complex rearrangements (mostly by MMBIR). However, with respect to NHEJ and MMEJ, the situation is different. Data on structural variation in the human genome suggest that a proportion of deletions of smaller sizes (≤100 kb) could result from NHEJ/MMEJ. On the other hand, mutations induced in mouse germ cells, following irradiation at G0/G1 phases, mostly derived from the repair of DSBs, predominantly via NHEJ, resulted in deletions of different sizes, some on the megabase scale. Although this discrepancy remains to be explained, our CNV dataset was largely comprised of structural variants without a flanking segmental duplication, 423/473 and 222/238 for case and control groups, respectively. The data favoured NHEJ and MMBIR as the two potential DNA repair mechanisms underlying the recovery of DSBs following human exposure to very low doses of ionizing radiation. Both mechanisms can mitotically generate genome rearrangements ranging from several megabases to a few hundred base pairs in size. Our findings were in agreement with^37,46,47^.

On the other hand, data from the present study indicated LCRs (SD) flanked 10.6% and 6.7% of the CNVs observed in the exposed and control groups, respectively. There was an increase of approximately 3 × in the fraction of induced CNVs harbouring SD among the children born to exposed parents, suggesting CNVs could also rise from NAHR, a potential mechanism for repairing DSBs. However, NAHR was not found to be the most relevant repair mechanism to fix DNA damage induced by low radiations doses in our cohort. Our findings are in agreement with^48^, who described the role of mutagenic agents in the formation DSB in the DNA, which in turn will lead to an increase in non-recurrent CNVs but not an increase in the occurrence rate of NAHR in the cells.

Arlt *et al*.^15^ used SNP array methodology to detected CNVs ranging in size from 2.7 kb to 34.2 Mb in fibroblasts exposed to IR. The authors reported that 1.5 to 3.0 Gy of ionizing radiation significantly induced somatic *de novo* CNVs in cultured human fibroblast cells with no difference in size distribution. In that study, the frequency of new CNVs increased in a dose-dependant manner and duplications were more frequently found. The results of the current study, reporting on germline mutations, were in disagreement with the aforementioned study as genomic losses were more frequent than genomic gais in both groups. On the other hand, our results were concordant with the report by^11^, who analyzed immediate and long-term effects of ionizing radiation on germline mutation in mice and found a significant increase in the frequency of CNVs in the offspring of exposed male mice. Animals incurred an absorbed dose of 0.45 Gy, generating 14 unique germline *de novo* CNV mutations, 12 were deletions and 2 were duplications.

Most germline mutations are paternally inherited due to the continuous nature of cell divisions in spermatogenesis throughout mitotic maintenance of spermatogonia in males as opposed to a few dozens cell divisions to form an oocyte in females^49^. Several studies have confirmed that the number of *de novo* mutations increases with paternal age. The linear relationship observed between paternal age and the number of mutations in the progeny is most often explained as a result of increased cell division, replication and DNA repair errors^40,50–54^. The current study also tested the hypothesis that males may contribute more to the burden of *de novo* CNVs in their offspring. We also found a positive relationship between the number of paternal meiosis at conception and *de novo* germline mutation rate in the progeny born to parents accidentally exposed to very low doses of ionising radiation in Goiânia (Brazil). We also confirmed that increased paternal age correlates positively to increased frequency of losses in the genomes of the offspring of both control and exposed groups. Among the cases, the extensive inter-individual variation observed in the rate of genomic losses obscured the effect of parental age. This finding may be explained by the individual dose of radiation absorbed by the fathers during the accident, which caused the induction of *de novo* CNVs in the offspring, confounding the effect of age of the exposed fathers on the observed mutation rate. In the current study, this finding in the control group is consistent with historical evidence that older men contribute more mutations to their children.

There has been a long-standing interest in the area of radiation protection and mutagenicity to declare ionizing radiation as a human germ cell mutagen. The current work was aimed to give some insights regarding the transmission of mutations through the germ line of the irradiated parents and we found it feasible to claim low doses of IR as a potent germline mutagen in humans.

## Conclusions

High density SNP array technology proved useful to retrospectively estimate the germline mutation rate in CNV in the offspring of a population accidentally exposed to very low doses of ionizing radiation. In the context of radiological protection, our data suggest that even doses of IR below 0.2 Gy incurred during whole-body exposure at lower dose rates could prove harmful by increasing the *de novo* mutation rate in the offspring. Thus, understating the genetic and biological consequences of exposure to IR has remained a long-standing goal for human populations, especially in the germ cell lines as the accumulation of additional mutations in the offspring of irradiated parents adds to the genetic risk for that particular population.The transgenerational effect of parental exposure to ionizing radiation has been well documented in animal models, focused on relatively large radiation doses and the conclusions have been extrapolated to humans^55^. However, little data on germline mutations has been generated on humans exposed to low absorbed doses of ionizing radiation. The mutation rate of *de novo* CNV, especially genomic losses, was proven to be a good biomarker of parental exposure to retrospectively study human populations exposed to low absorbed doses of IR as reported here.

## Materials and Methods

### Sampling Groups

The exposed group was comprised of 12 families of which at least one parent was directly exposed to ionizing radiation (IR) of Caesium-137. This group included 40 people: 12 mothers, 12 fathers, and 16 children conceived after the accident. All those exposed people incurred absorbed doses of ≤0.2 Gy, mostly due to whole-body external exposure to gamma IR. For extensive review on dose estimates for the Goiânia population, please refer to IAEA’s report on the accident^2^. The cut-off at 0, 2 Gy was selected as the lowest absorbed dose detected by traditionally used genetic biomarkers, such as micronuclei and chromosome aberrations, as the biological effect of very low doses of IR has remained unclear and inconsistent in human populations. Moreover, discrepant information has been generated regarding the induction of germline mutations in the offspring of exposed parents. Additionally, ≤0.2 Gy included the smallest absorbed doses recorded for the Goiânia population based on biodosimetry using dicentric chromosomes. A group of people with no history of occupational, accidental or medical exposure to IR participated as a control population from Goiânia. The control group comprised of 8 trios (24 people), corresponding to a mother, a father, and one biological child from the Goiânia population who voluntarily agreed to participate in the study. For both cases and controls, biological paternity and maternity were confirmed to an index of 99.99%.

A total of 10 mL of peripheral blood was withdrawn from all participants who voluntarily agreed to take part in the study. DNA was isolated from each sample and stored at −20 °C. Participants answered a lifestyle questionnaire and signed an informed consent from according to the national ethical guidelines for research with humans. All participants, including mothers, fathers, and children (for minors consent was signed by the legal guardian) voluntarily signed the informed consent forms approved by the Ethics Committee on Human Research from the Pontifical Catholic University of Goiás (CEP-PUC Goiás), kept under the registration numbers CAAE-205000.073183/2004-45, and CAAE-49338615.2.0000.0037/2016. The study was carried out at Núcleo de Pesquisas Replicon from PUC Goiás having all methods and procedures carried out in accordance with relevant international guidelines and regulations. Moreover, the CEP-PUC Goiás also approved the experimental protocols.

### DNA Extraction, Isolation, and Quantification

DNA extraction and isolation was carried out using DNA Illustra Blood GenomicPrep® Mini Kit (GE Healthcare, USA) following the instructions provided by the manufacturer. DNA concentration was spectrophotometrically estimated in a NanoVue^®^ Plus (GE Healthcare Life Sciences, United Kingdom).

### Chromosomal Microarray Analysis (CMA)

CMA was performed with the GeneChip^®^ CytoScanHD^TM^ (Affymetrix, USA) following the manufacturer’s recommendations without modifications. Capture and digitalization of fluorescent signals were done with the aid of the GeneChip^®^ Command Console^®^ software (AGCC^®^, Affymetrix, USA). Chips that met the quality control metrics, namely *Median Absolute Pairwise Difference* (MAPD) e SNP-QC, with parameters set at de MAPD ≤ 0.25 e de SNP-QC ≥ 15, had their data included in the analyses.

Chromosomal analyses were performed using ChAS^®^ (Affymetrix, USA). Filters were set at 8 and 15 SNP markers for microdeletion and microduplication calling, respectively. A *de novo* CNV was considered if the mean distribution of markers was ≤2 kb, CNV’s size were set as ≥1 kb, and median log2 ratio ±0.40. Whenever a CNV was found with a chance to be imputed, based on the distance of its markers, that segment was manually cut to adjust the area around the markers, following the rules for marker counts and their mean distribution along the DNA segment. In the current study, only *de novo* CNVs were scored. Sex chromosomes were not included in the analysis. Using the mendelian error rate and the graph table from ChAS^®^ (Affymetrix, USA), the parent of origin (POR) for each CNV was assessed.

### Germline Mutation Rate Estimates (MR_cNV_)

The rate of *de novo* CNV was estimated taking into consideration the formation of CNV/locus/generation. The MR_CNV_ was estimated applying the following equation 1 (6, 7):

1 $${{\rm{MR}}}_{{\rm{CNV}}}=\,\frac{\sum {{\rm{T}}}_{{\rm{CNV}}}}{{\rm{\varepsilon }}!\times {\rm{a}}\times {\rm{shg}}}$$

where:

ΣT_CNV_: Total size o f CNVs (bp)

ε!: Number of meiosis (2)

a: Bialelic locus (2)

shg: Size of haploid genome (2.9 × 10^9^), according to the genome build GRCh37/hg19.

Equation 1 was applied to both CNVp (loss) and CNVg (gain). The MR_CNV_ corresponded to the total mutation rate, referred to in the text as CNV burden, which pertains to the sum of MR_CVN_ of gain and MR_CNV_ of losses in a given genome.

In order to estimate the number of paternal meiosis for both cases and controls, we followed Equation 2, based on the age of father at the time of child conception:

2 $${\rm{No}}.\,{\rm{\rho }}!=((({\rm{PA}}\times {\rm{m}})-({\rm{dp}}+{\rm{A}}))\times {\rm{d}}/{\rm{m}})\times {\rm{t}}{\rm{\rho }}!{\rm{d}}$$

where:

No. ρ!: Number of paternal meiosis

PA: Paternal age (years)

m: Months/year

dp: Duration of pregnancy (months)

A: Average age for starting sperm production in humans [12 year-old] (months)

d/m: Total days in a month.

tρ!d: Average of the total number of meiosis per day in human males (~25 × 10^6^).

### Segmental Duplication Flanking CNVs

The occurrence of segmental duplications (SD) around the annotated CNVs was carried out doing active search in the databank from UCSC Genome Browser (http://www.genome.ucsc.edu). In order to associate a CNV to a SD we considered all surrounding DNA sequences up to 100x the size of a particular CNV. SD flanking a CNV must have met the criteria of similarity ≥90%.

### Statistical Analysis

The non-parametric test of Mann-Whitney U was applied to estimate the differences in mutation frequencies, size of CNVs, MR_CNV_/chromosome, number of markers and genes for losses, gains and burden, when comparing cases and controls. MR_CNV_ did not follow a normal distribution according to the Shapiro-Wilk’s test. Spearman’s rank correlation coefficient was applied to analyse the effect of paternal meiosis at conception on the burden of CNV mutation. The binomial test was used to test the differences between cases and controls with respect to the frequencies of CNVloss and CNVgain, and the frequency of flanking SD in CNVs. Discriminant function analysis was used to predict the contribution of the variables (predictors): number of paternal meiosis, frequency of CNVloss and CNVgain, mean parental age at conception, number of markers in a CNV, number of genes in a CNV, and size of CNVs (kb) in discriminating cases and controls. In these cases, exposed and control groups were used as the categorical dependent variables. We took into consideration the 3 predictors that had the largest contribution to discriminate the groups in order to calculate the coefficient for the discriminating function. The post-hoc χ^2^ test was used to examine the differences in the frequency of CNVs classified in categories according to their size and number of SNP markers. Also, Chi square was used to test for the distribution in CNVs in chromosomes in order to validate potential hotspots for both genomic losses and genomic gains for case and control groups. In order to compare the contribution of the parental exposure and the parent of origin (POR) for the CNVs on the burden of mutations found in the current study, Two-way ANOVA followed by Tukey’s test *a posteriori* were performed. All statistical analyses were performed using SPSS^®^ 23.0 at a level of significance of 5% (p < 0.05).

### Data availability Statement

All relevant data are within the paper.
